# Factitious Conjunctivitis: A Great Imposter With Compelling Evidence

**DOI:** 10.7759/cureus.98147

**Published:** 2025-11-30

**Authors:** Shahrina Mahfooz, Sharah Rahman, Tarzia Asma Zafrullah, Rodela Saha, Mohammad Ibn Abdul Malek

**Affiliations:** 1 Pediatric Ophthalmology and Strabismus, Bangladesh Eye Hospital and Institute, Dhaka, BGD; 2 Cornea and Refractive Surgery, Bangladesh Eye Hospital and Institute, Dhaka, BGD; 3 Ophthalmology, Lions Eye Institute and Hospital, Dhaka, BGD; 4 Ophthalmology, Moorfields Eye Hospital, Bedford, GBR

**Keywords:** conjunctival biopsy, factitious conjunctivitis, pseudomembranous conjunctivitis, psychiatric disorder, self-induced conjunctivitis

## Abstract

Factitious illnesses are conditions in which a patient consciously produces symptoms or outward manifestations of ill health. Although uncommon, ophthalmologists should be mindful of the risk of self-inflicted ocular injuries, especially while caring for patients with psychiatric problems. Identifying ocular factitious lesions that only affect the conjunctiva can be difficult. An acceptable index of suspicion, non-judgmental confrontation, and psychiatric consultation promote a successful clinical approach. Making a diagnosis sometimes requires conjunctival biopsies. Focusing on any potential behavioural anomalies and referral to a psychiatrist are also helpful. The authors describe an 16-year-old who self-inoculated rust particles inside the conjunctiva of her right eye, sparing her cornea, in this case report.

## Introduction

Patients with factitious disorder, also known as Munchausen syndrome, deliberately produce or feign physical or psychological symptoms to assume the sick role and receive medical attention [1]. This condition represents a complex psychiatric challenge in which individuals may harm themselves for subconscious psychological gain rather than external incentives [2]. In ophthalmic practice, factitious ocular disorders are uncommon but well-documented and can present as self-inflicted conjunctivitis, keratitis, corneal ulcers, or even chemical burns [3]. These patients may claim accidental trauma, exaggerate minor symptoms, or intentionally introduce foreign materials into the eye to simulate disease [4].

We present a case involving a young female patient who deliberately inserted rust particles into the inferior conjunctival fornix of her right eye, resulting in marked hyperemia, discharge, and discomfort mimicking bacterial or chemical conjunctivitis. Careful history-taking and slit-lamp examination, coupled with the absence of objective findings correlating with the severity of symptoms, led to the suspicion of a factitious disorder. This represents, to the best of our knowledge, the first documented instance of rust particle-induced factitious conjunctivitis. Early recognition of such presentations is crucial to prevent unnecessary interventions and to ensure appropriate psychiatric referral and multidisciplinary management [5].

## Case presentation

**Figure 1:**
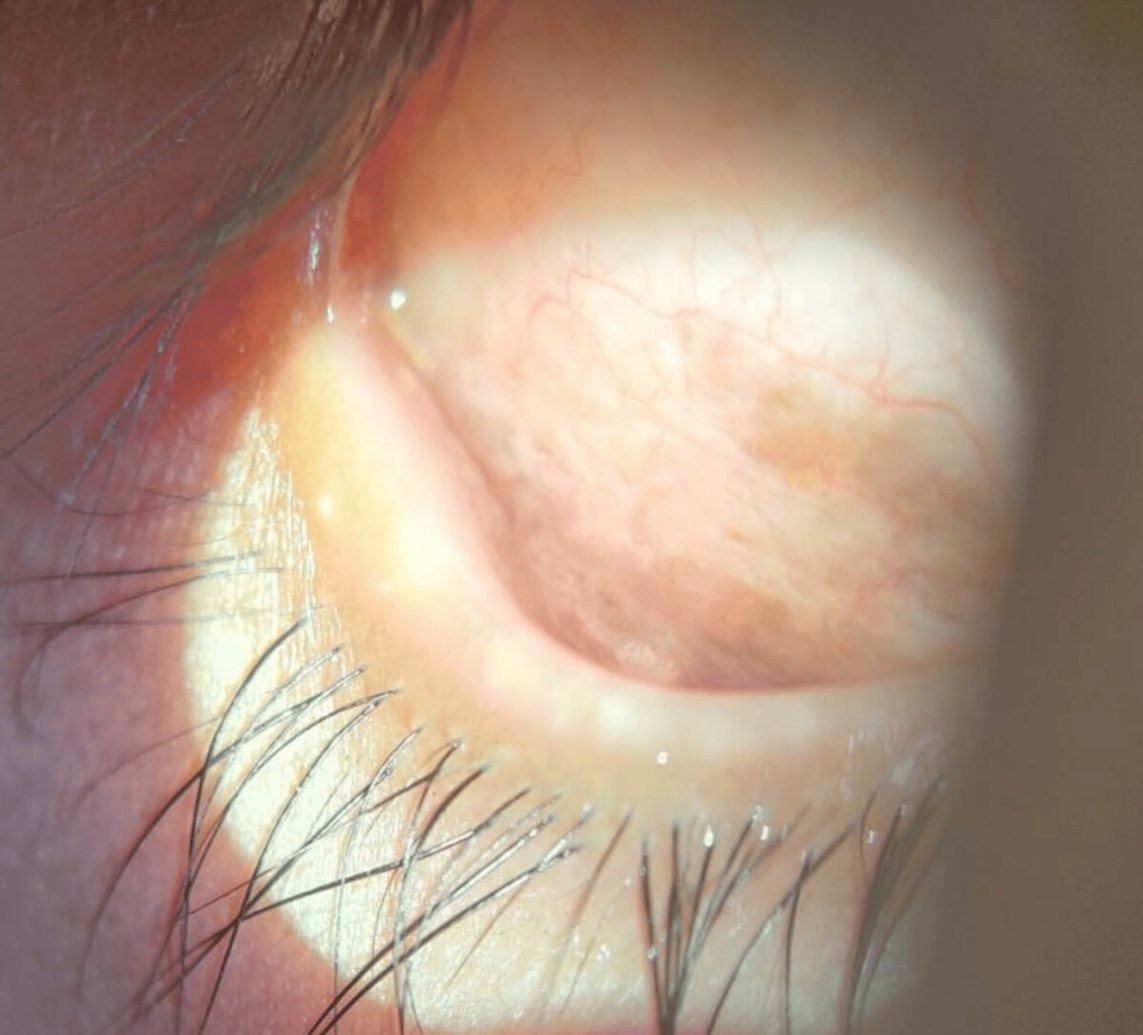
Diffuse muddy discolouration observed in the inferior conjunctival fornix

A local ophthalmologist referred a 16-year-old girl as a probable case of unilateral chronic pseudomembranous conjunctivitis unresponsive to topical antibiotics and steroids. At presentation, her unaided visual acuity was 20/20, N6 in both eyes. Examination of the right eye (OD) revealed a reddish-brown discolouration of the lateral inferior fornix, while the left eye appeared normal (Figure 1).

**Figure 2:**
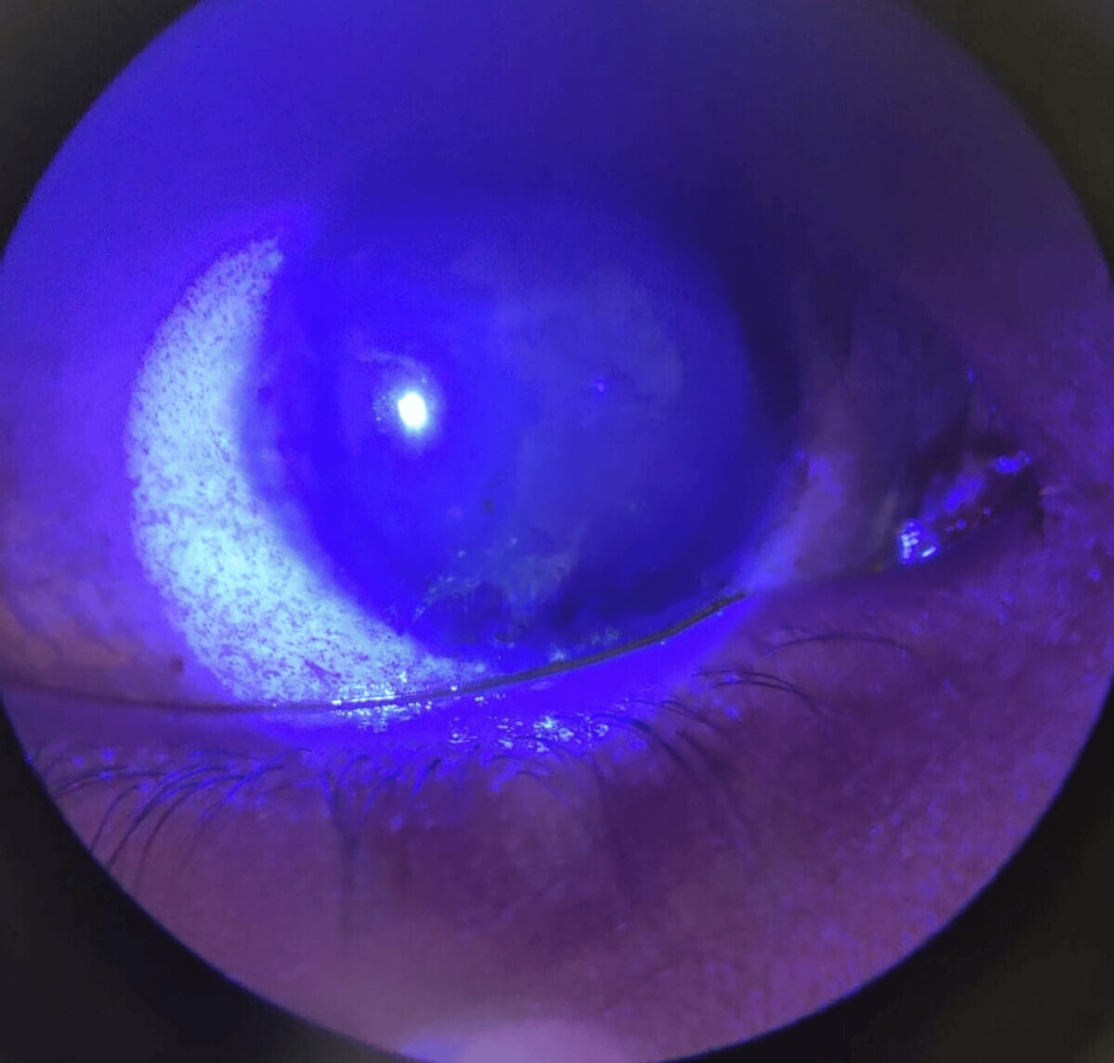
Superficial punctate keratitis in the inferior cornea

Apart from a few follicles and mild fornicial congestion, there were no signs of active inflammation or actual membrane formation. The remainder of the conjunctiva was unremarkable. Fluorescein staining demonstrated a devitalised area in the lateral inferior fornix, and there was superficial punctate epithelial keratitis in the inferior cornea (Figure 2).

**Figure 3:**
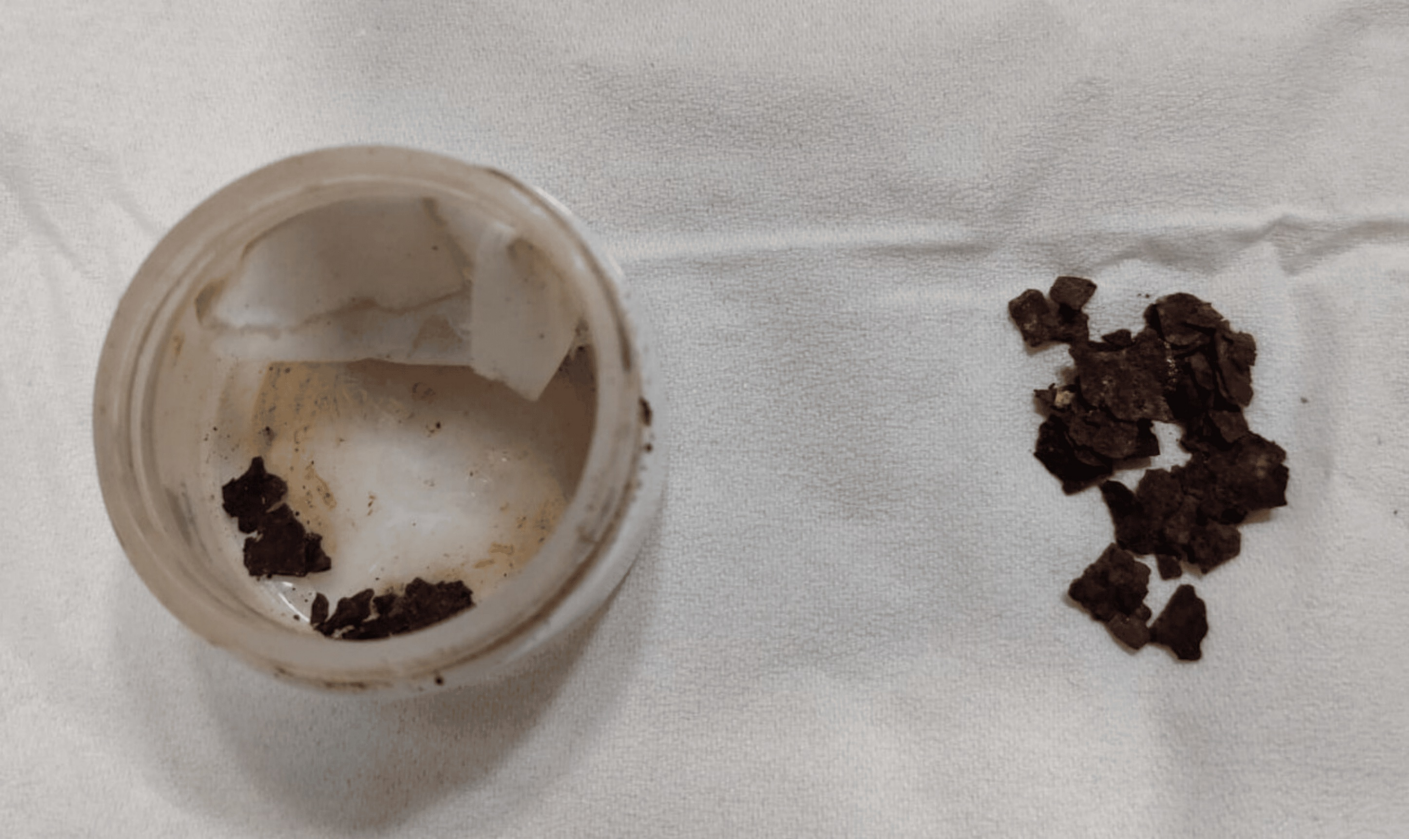
Rust particles collected from the inferior fornix

On inquiry, the patient's father reported that each morning she complained of irritation and claimed a membrane had formed in her right eye overnight. Her parents had been collecting the expelled material, identified as rust particles, and presented them during the visit (Figure 3). They lived in a tin-roofed rural house, where such rust (iron oxide) commonly accumulates due to the oxidation of the metal surface.

Separate interviews with the parents revealed months of unusual behaviour and inconsistent symptom chronology. The differential diagnosis included allergic or immune-mediated conjunctivitis, toxic or chemical conjunctivitis, and dermatologic causes such as atopic dermatitis, all of which can present with recurrent ocular redness. Psychiatric or behavioural disorders were also considered, as the pattern and persistence of symptoms raised concern for possible self-inflicted ocular injury. However, the lesion's confinement to the right inferior fornix, corresponding to her dominant hand, and the absence of matching inflammatory signs strengthened the suspicion of self-inflicted injury. The final diagnosis of factitious conjunctivitis secondary to rust particle insertion was made. To establish a definitive diagnosis, a conjunctival biopsy was recommended, but the parents declined any invasive procedure. As a result, the diagnosis was made based on clinical evaluation. The patient received psychological counselling and was referred to a psychiatrist for further evaluation. Topical lubricants and prophylactic antibiotics were prescribed to prevent secondary infection. Unfortunately, she was lost to follow-up, a frequent occurrence in cases involving self-inflicted ocular injury.

## Discussion

Factitious conjunctivitis is an uncommon but clinically significant disorder in ophthalmology, where patients deliberately induce or simulate ocular inflammation to assume the sick role rather than gain external benefits [1]. Such self-inflicted ocular conditions have been described under the broader term Munchausen syndrome by proxy or factitious disorder imposed on self [2]. Reported foreign materials include cotton-wool fibres, freshly scraped dental plaque, razor blades, and needle-like objects introduced into the conjunctival sac to mimic chronic conjunctivitis [3,4]. In our case, the patient used rust particles collected from her tin-roofed home, representing a novel mechanism of self-inflicted injury.

Clinically, factitious ocular disease is challenging to diagnose due to its inconsistent symptoms, its dramatic presentations, and the absence of findings that match the patient's complaints [5-7]. The discrepancy between severe subjective symptoms and mild or localised objective signs is a hallmark. In our patient, unilateral involvement of the right inferior fornix, corresponding to her dominant hand, and the minimal inflammatory reaction were key diagnostic clues. Similar laterality-based self-inflicted patterns have been documented by Al-Faky, where the dominant hand dictated the side of injury [5].

Factitious lesions may affect various ocular structures, including the conjunctiva, cornea, and eyelids, with potential for secondary infection or scarring [8,9]. If untreated, repeated trauma may lead to corneal thinning, ulceration, or visual impairment [10]. A careful slit-lamp examination, supported by fluorescein staining, can help identify devitalised epithelium or recurrent mechanical trauma. However, diagnosis depends primarily on maintaining clinical suspicion and performing a tactful, non-confrontational history [6].

The management of factitious ocular disorders necessitates a multidisciplinary approach, striking a balance between ocular treatment and psychological care and support. A non-judgmental and empathetic attitude helps build rapport and prevents further self-harm. Early psychiatric referral is essential to address the underlying behavioural or emotional distress that sustains the disorder [7,10]. As in our case, psychological counselling was advised, though the patient was lost to follow-up, a common outcome described in previous reports, reflecting patients' reluctance to engage once the psychogenic origin is discussed [11].

Factitious ocular disease remains one of the most difficult entities to recognise in ophthalmic practice due to its core features of deception and denial [12]. A detailed, tactful history may uncover hidden psychosocial stressors, while a compassionate clinical attitude fosters cooperation and rehabilitation. Increasing awareness of factitious conjunctivitis among ophthalmologists and the public is vital for early recognition, the prevention of unnecessary interventions, and the reduction of vision-threatening complications.

## Conclusions

Factitious ocular disease, characterised by deception, denial, and intentional symptom fabrication, represents a significant diagnostic challenge in ophthalmic practice. Any persistent redness that cannot be associated with a clinical diagnosis should arouse suspicion of factitious conjunctivitis. A thorough and detailed examination of history can reveal the psychological distress that motivates such behaviour. An empathetic, non-judgmental approach is essential, as it helps build trust, encourages honest disclosure, and reduces the risk of further self-harm. In such cases, counselling for both the patient and the family plays a crucial role in achieving effective management. Timely psychiatric referral facilitates thorough management that encompasses both ocular and psychological dimensions. This case emphasises the importance of collaboration between ophthalmologists and mental health professionals for accurate diagnosis and comprehensive care. 
